# Anxiety and disease awareness in individuals with heredity for abdominal aortic aneurysm

**DOI:** 10.1177/09691413241278224

**Published:** 2024-09-09

**Authors:** Nina Fattahi, Olga Nilsson, Sverker Svensjö, Joy Roy, Anneli Linné, Rebecka Hultgren

**Affiliations:** 1 Department of Clinical Science and Education, 27106 Karolinska Institutet at Södersjukhuset, Stockholm, Sweden; 2 Section of Vascular Surgery, Department of Surgery, 59570 Södersjukhuset AB, Stockholm, Sweden; 3 Stockholm Aneurysm Research Group, STAR, Department of Molecular Medicine and Surgery, 27106 Karolinska Institutet, Stockholm, Sweden; 4 Department of Vascular Surgery, 59562 Karolinska University Hospital, Stockholm, Sweden; 5 Department of Surgical Sciences, 8097 Uppsala University, Uppsala, Sweden; 6 459413 Department of Surgery, Centre for Clinical Research Dalarna, Falun, Sweden

**Keywords:** Abdominal aortic aneurysm, heredity, first-degree relatives, health-related quality of life, family history, anxiety

## Abstract

**Objective:**

The psychological consequences of being aware of an increased risk of developing abdominal aortic aneurysm as a first-degree relative of a person with abdominal aortic aneurysm are hitherto unexplored. This study investigates the awareness of heritability and anxiety in male and female adult offspring of abdominal aortic aneurysm patients compared to controls. Health-related quality of life among participants with aortic pathology was compared to participants with normal aortic diameters.

**Methods:**

This was a cross-sectional point prevalence study based on the participants examined in the Detecting Abdominal Aortic Aneurysm in First Degree Relatives Trial (DAAAD; 752 adult offspring, 756 matched controls), 2020–2022. Questionnaires about health-related quality of life and study-specific questions regarding awareness of heritability were collected prior to the aortic ultrasound.

**Results:**

Attendance rate was higher among individuals with heredity compared to controls (67% vs. 52%, *p* < 0.001). Of 1508 adult offspring examined, 65% reported having a close relative with abdominal aortic aneurysm (6% in controls). Female adult offspring reported higher awareness of heritability than controls (38% vs. 12%, *p* < 0.001), as did males (32% vs. 8%, *p* < 0.001). A slight majority of participants with awareness reported anxiety (54% of female offspring; 51% of male). There were no measured differences in health-related quality of life between the groups when standard health-related quality of life instruments were used.

**Conclusion:**

The higher-than-expected proportion of adult offspring with awareness of heritability and anxiety about such risk indicates that we fail to communicate risk to this group appropriately via the current channels of information within the healthcare system. This calls for the development of dedicated strategies for improved communication of abdominal aortic aneurysm risk to patients and their next of kin.

## Introduction

The hereditary patterns of abdominal aortic aneurysm (AAA) have previously been reported to carry a higher risk for disease development and potentially also introduce a different disease progression.^1–5^ It remains unclear whether the familial risk of AAA development is associated with negative psychological effects in first-degree relatives (FDRs). It is methodologically challenging to invite and examine adult offspring of AAA patients regarding heritability within conventional care trajectories for AAA patients, due to the high mean age at development of the disease.

National screening programs for AAA have been successfully implemented, leading to a significant reduction in aneurysm ruptures and related deaths.^6,7^ The possible negative effects of AAA screening and diagnosis on health-related quality of life (HRQoL) have been evaluated in different contexts showing somewhat diverging results, with both immediate decrease in HRQoL at diagnosis, which is recovered within a year, and lack of alterations in HRQoL.^8–11^ The psychological impact of screening and diagnosis has been described in breast cancer and colorectal screening programmes, with elevated anxiety and depression symptoms initially which normalise in the long-term follow-up.^12,13^ HRQoL may also be influenced by the timing of data collection and the length of the follow-up period, and therefore it is important to collect this information prior to a possible diagnosis. It has been reported that individuals who are diagnosed later with AAA have poorer general wellbeing before being aware of their AAA, compared to individuals without the disease.^11,14^

In individuals with hereditary cancer syndromes, a higher degree of anxiety and depression has been reported.^15^ Whether individuals with a hereditary risk for AAA experience negative psychological effects is to date unknown. In the recently published results from the DAAAD study (detecting abdominal aortic aneurysm in adult offspring to AAA patients, *n* = 1500), exploring the proportion of aortic diameters exceeding 25 mm in adult offspring to AAA patients, a higher attendance rate was found in adult offspring than matched controls without heredity. This could indicate a higher awareness of their heredity, rendering them more prone to participate in the study.^16^

The primary aim of this study was to investigate the awareness of heritability for AAA and possible anxiety in male and female adult offspring of AAA patients compared to matched controls. The second aim was to explore whether this anxiety and awareness could be measured by validated HRQoL instruments. The third aim was to estimate the HRQoL among participants who were later diagnosed with aortic pathology compared to those with normal aortic diameter.

## Methods

### Study cohort and design

This is a cross-sectional study in accordance with the STROBE guidelines for observational studies.^17^ The study cohort was examined in the DAAAD trial (Detecting abdominal aortic aneurysm in adult offspring to AAA patients) 2020–2022, which explored the prevalence of AAA (≥30 mm aortic diameter) or sub-aneurysmal aorta (SAA) (≥25 mm) in the adult offspring of diagnosed AAA patients compared to a matched control population without heredity.^16^ These pathologies (AAA, SAA) are defined as any aortic pathology (AP). The study design was reported in a methodology paper in 2022 and the basic aortic ultrasound findings and details of the cohorts have been published.^16,18^

Through the Swedish National Patient Register (NPR), 69,000 individuals with AAA (born 1900–1953, index person) were identified in September 2020 by the National Board of Health and Welfare (NBHW). The adult offspring of these individuals with an address in the Stockholm Region were identified and extracted through the Multigeneration register. The control population was matched by age and sex and identified through Statistics Sweden. The Region of Stockholm covers 6514 km^2^ with a mixed urban and rural setting. The matching of controls regarding the mentioned factors would at least partly increase the possibility that the control group is similar to the adult offspring group regarding habitation (rural/urban) and socioeconomic status. A random selection was performed at the NBHW. Invitations to participate were sent out through a random selection in the SPSS program among 3800 individuals.^18^ Information on the individual's specific risk or heredity was not disclosed. The invitation contained basic information on the intention of the project, a pre-booked ultrasound appointment, a consent form, and a personal code to access the web-based questionnaires. All included persons had answered questionnaires regarding their general health, awareness of AAA disease, and possible anxiety regarding the risk for AAA disease prior to the ultrasound examination of the infrarenal aorta. The web-based questionnaires were filled out at home, or on a tablet at the examination site, with hands-on support by clinical staff if requested. Between November 2020 and June 2022, 2585 invitations were sent out in order to reach the total designated inclusion of 1508 participants, comprising both women and men, adult offspring of AAA patients, and matched controls.

## Questionnaires

Two validated questionnaires were used, the Hospital Anxiety and Depression Scale (HADS) and EuroQol Five Dimension Scale (EQ-5D, with five levels), along with study-specific questions regarding awareness and anxiety about the hereditary risk of disease.^18^

HADS is a validated self-reported questionnaire that aims to measure symptoms of anxiety (HADS-A) and depression (HADS-D), during the past week. It includes 14 items (7 pertaining to anxiety and 7 to depression). A cut-off score of 8 or more for anxiety has a specificity of 0.78 and a sensitivity of 0.9 and for depression, a specificity of 0.79 and a sensitivity of 0.83.^19,20^ Scores of 0–7 are within the normal range, 8–10 mild, 11–14 moderate and 15–21 indicate severe anxiety or depression.^19,20^ HADS has previously been used in studies regarding screening of men.^10,14^

The 5-level EQ-5D is a standardized instrument to measure HRQoL. It includes five dimensions (mobility, self-care, usual activities, pain/discomfort and anxiety/depression). Each of these takes one of five responses reflecting severity (no/slight/moderate/severe and extreme problems). It provides a single index value of the current HRQoL. Additionally, a health status measurement is obtained through a self-rating on a thermometer that ranges from 0 to100.^21–23^

The two study-specific questions on awareness were validated through cognitive interviews with four males and four females in the age range 50–80 years with either aortic pathology (*n* = 3) or normal aortic diameter (*n* = 5) (see Supplemental Table S1). All questionnaires were filled out before the ultrasound examination.

## Sample size

The sample size was primarily estimated in the DAAAD cohort for the prevalence of AAA in adult offspring versus matched controls, which resulted in 1500 examined participants.^18^ The secondary outcome was awareness of heredity among the adult offspring. In siblings, it has previously been reported that 11% of them had knowledge regarding heredity leading to an ultrasound examination.^3^ It is probable that adult offspring of AAA patients have a higher awareness of the possible hereditary traits compared to siblings due to knowledge of disease in parents. This field has never been studied nor has the degree of awareness regarding AAA risk in the general population; therefore estimations of awareness among 15% of offspring and 5% of the control group generated a sample size calculation for a minimum of 140 participants in each treatment arm, with alfa 0.05, beta 0.2 and power 0.8.

## Outcome

The primary outcome was awareness regarding the hereditary risk of developing AAA disease for male and female adult offspring of AAA patients as compared to the general population. The secondary outcome was anxiety regarding the hereditary risk. The third outcome was possible differences in anxiety levels using EQ-5D-5L and HADS between adult offspring of AAA patients and matched controls for women and men separately. The fourth outcome was the general HRQoL among individuals identified with aortic pathology as compared to individuals without the disease.

## Statistical analysis

All participants’ self-reported risk factors and baseline characteristics were described with descriptive statistics; and adult offspring and controls separately. The data were tested for normality using the Shapiro-Wilks test, which indicated that the data were not normally distributed. Given the large sample size, the assumption of normal distribution is still valid. The data were analysed both with t-test and non-parametric test Mann-Whitney *U* and there was no difference in significance levels. Continuous variables were reported by mean and standard deviation, and differences were tested by *t*-test. Categorical data were reported in proportions and statistical significance was tested with the chi-square test or Fisher's exact test where appropriate. The awareness was reported in proportions for women and men separately, and statistical significance was tested with the chi-square test. Subgroup analyses were performed between male and female adult offspring. Additionally, analysis of individuals with AP in comparison to normal aortic diameter was conducted using nonparametric tests (Mann-Whitney *U*).

The study was approved by the Swedish Ethical Review Authority (Dnr: 2019-01076) and all participants provided written informed consent to participate. Trials registration: ClinicalTrials.gov identifier, NCT4623268.

## Results

### Baseline characteristics and participation

Overall, 1508 participants were included, 809 women (402 adult offspring; 407 controls) and 699 men (350 adult offspring; 349 controls). Most characteristics were similar when comparing adult offspring to controls (Table 1). The mean age for adult offspring and controls was similar (64.1 (SD 7.7) vs. 64.3 (SD 8.0)). No difference was noted for smoking habits, hypertension, and lipid-lowering therapy. The total number of participants with aortic pathology (≥ 25 mm) was 22, of which five were women and 17 were men (Table 1). The participation rate differed among adult offspring and controls, 752/1126 (67%) and 756/1459 (52%), respectively (*p* < .000).

**Table 1.** Characteristics of the 1508 participants in the study, including a number of cases with aortic pathology. Information regarding smoking and comorbidity is collected by self-reported questionnaires.

	Adult offspring (*n* = 752)	Controls (*n* = 756)	*p*-value
Men, *n* (%)	350 (47%)	349 (46%)	
Age, mean years (SD)	64.1 (7.7)	64.4 (8.0)	0.551
Height, mean cm (SD)	173.1 (10.5)	172 (9.8)	0.034
Weight, mean kg (SD)	79.4 (17)	77.8 (15.1)	0.059
BMI, mean kg/m^2^ (SD)	26.6 (7.8)	26.2 (4.2)	0.216
Abdominal aortic diameter, mean mm (SD)	17.6 (3.4)	17.1 (3.0)	0.008
Aortic pathology/diameter ≥ 25 mm	15 (2.0%)	7 (0.9%)	0.090
Smoking status			0.251
- Never	61 (8%)	45 (6%)	
- Ever (former/current)	687 (92%)	710 (94%)	
Hypertension	310 (41.4%)	292 (38.7%)	0.292
Diabetes mellitus	70 (9.3%)	71 (9.4%)	0.969
Angina pectoris	21 (2.8%)	34 (4.5%)	0.077
Heart failure	22 (2.9%)	33 (4.4%)	0.136
Kidney failure	11 (1.5%)	12 (1.6%)	0.844
Pulmonary disease	55 (7.3%)	53 (7.0%)	0.825
Antihypertensive treatment	290 (38.8%)	296 (39.3%)	0.847
Lipid-lowering therapy	173 (23.3%)	151 (20.1%)	0.136
Anti-platelet therapy/anticoagulant medication	106 (14%)	126 (17%)	0.166
Other medications	360 (49.8%)	361 (50.1%)	0.895

SD: standard deviation; BMI: body mass index.

### Knowledge of family history

Overall, 490/752 (65%) of adult offspring (female and male) were aware that they had a close relative with AAA while 164/752 (22%) did not have knowledge about their relative's disease (see Supplemental Table S2). The majority of the female adult offspring responded that they had a close relative with AAA (290/402, 72%), while 81/402 (20%) stated that they did not know (Table 2). Similar results applied to the male adult offspring (Table 2). Most male adult offspring reported a close relative with AAA (200/350, 57%), while 83/350 (24%) did not know (Table 2). Among persons with a close relative with AAA, 142/290 (49%) of the female adult offspring and 94/200 (47%) of the male stated that the relative was diagnosed with an acute aneurysm incident (Table 2).

**Table 2.** The proportions of examined female and male offspring and matched controls reporting their knowledge of next-of-kin with aneurysm disease, as well as their awareness and anxiety about the hereditary risk.

Question 2	Yes	No	Don’t know	Yes	No	Don’t know	*p*-value
Were you aware of the risk to develop AAA as an FDR?	151 (38%)	204 (51%)	44 (11%)	51 (12%)	300 (74%)	56 (14%)	0.001
If yes: Have you experienced anxiety for such risk?	81 (54%)	64 (43%)	6 (4%)	10 (20%)	37 (73%)	4 (8%)	0.001
If yes: Have you thought about how you could deal with this risk or anxiety?	51 (35%)	78 (53%)	17 (12%)	14 (27%)	29 (57%)	8 (16%)	0.577

Men	Adult offspring *n* = 350			Controls *n* = 349			
Question 1	Yes	No	Don’t know	Yes	No	Don’t know	p-value
Do you have a close relative with AAA?	200 (57%)	66 (19%)	83 (24%)	17 (5%)	186 (53%)	145 (42%)	0.001
If yes: Did this person have an acute aneurysm incident (rupture) caused by this diagnosis?	94 (47%)	79 (40%)	27 (13%)	11 (65%)	6 (29%)	1 (6%)	0.432

Question 2	Yes	No	Don’t know	Yes	No	Don’t know	*p*-value
Were you aware of the risk to develop AAA as an FDR?	110 (32%)	184 (53%)	53 (15%)	27 (8%)	235 (68%)	83 (24%)	0.001
If yes: Have you experienced anxiety for such risk?	56 (51%)	54 (49%)	1 (1%)	3 (11%)	22 (78%)	3 (11%)	0.001
If yes: Have you thought about how you could deal with this risk or anxiety?	45 (41%)	58 (53%)	7 (6%)	4 (14%)	22 (79%)	2 (7%)	0.019

AAA: abdominal aortic aneurysm; FDR: first-degree relative.

### Awareness and anxiety

Among the whole adult offspring cohort of 752 individuals, 35% reported awareness of their hereditary risk for AAA and 18% had experienced anxiety (81/402, 20% female and 56/350, 16% male adult offspring) (Supplemental Table S2). The female adult offspring reported a higher awareness of heritability than controls (151/402, 38% vs. 51/407, 12%, *p* < 0.001). The corresponding proportions were similar in the male group (Table 2).

**Figure 1.**
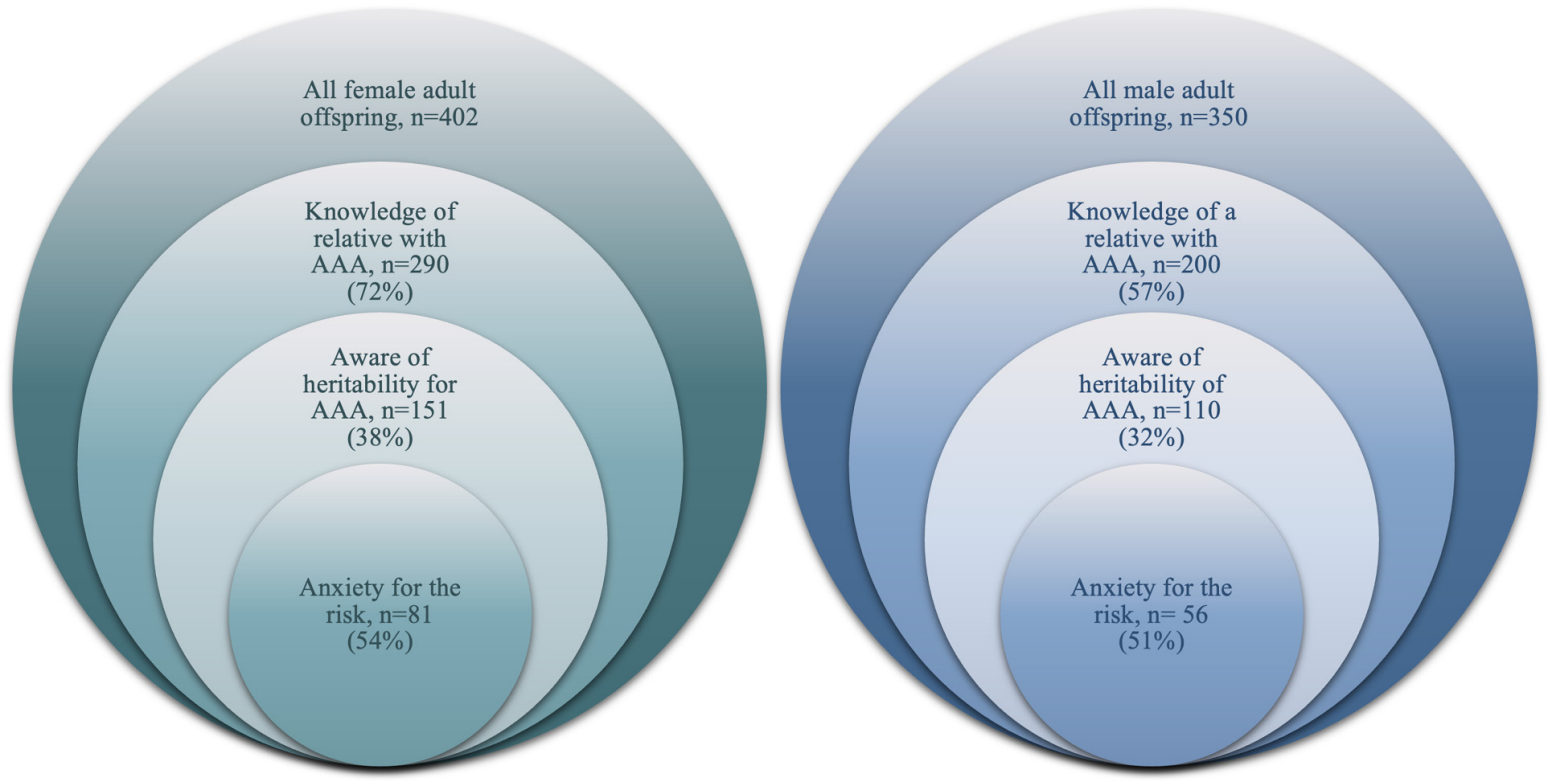
The proportion of adult offspring, female and male, with awareness and related anxiety, indicated by responses to study-specific questions.

More than half of female and male adult offspring with awareness of heritability also reported anxiety relating to this risk (81/151, 54% and 56/110, 51%, respectively) (Figure 1).

A majority of both female and male adult offspring with reported anxiety had a close relative who had experienced an acute aneurysm incident (47/81, 58% and 34/56, 61%, respectively).

### HADS/EQ-5D

When comparing adult offspring and controls with no consideration of sex, total mean HADS (*p* = 0.039) and HADS-D (*p* = 0.031) were significantly higher for controls (Supplemental Table S2). No difference was noted when considering the results of the HADS-A/D score ≥ 8, which also applied to the EQ-5D visual analogue scale (VAS) and the EQ-5D index score (Supplemental Table S2). There was no difference between the female adult offspring and controls regarding the overall assessment of HADS and EQ-5D (Table 3). The male controls stated a higher general level of HADS-A, HADS-D and total mean of HADS compared to male adult offspring (Table 3).

**Table 3.** Female and male adult offspring and matched controls reporting anxiety and depression symptoms via the HADS and EQ-5D instruments. A higher HADS score (≥8) is considered as indication of anxiety (A) or depression (D). A higher score on the EQ-5D scale indicates better well-being.

Men	Adult offspring *n* = 350	Controls *n* = 349	
HADS total, mean (SD)	5.4 (5.3)	6.4 (6.0)	0.023
HADS-A, mean (SD)	3.1 (3.3)	3.6 (3.5)	0.050
HADS-D, mean (SD)	2.3 (2.6)	2.8 (3.1)	0.028
HADS-A score ≥ 8	37 (11%)	46 (13%)	0.294
HADS-D score ≥ 8	15 (4%)	24 (7%)	0.141
EQ-5D index score, mean (SD)^a^	6.4 (2.0)	6.7 (2.3)	0.157
EQ-5D VAS 0–100 (SD)	79.2 (15.0)	78.8 (15.8)	0.714

HADS: Hospital Anxiety and Depression Scale; EQ-5D: EuroQol Five Dimension Scale; VAS: visual analogue scale; SD: standard deviation.

^a^EQ-5D: min 5, max 25.

Among the 137 adult offspring reporting anxiety in the study-specific questionnaire reported above, 108 (79%) could not be captured through the generic HADS questionnaires (HADS-A score ≤ 8).

### Sex differences

Female adult offspring had knowledge of their relatives’ AAA disease to a larger extent than men (290/402, 72% vs. 200/350, 57%, *p* = 0.001) (Table 4). No distinct sex differences were observed regarding awareness of heritability (151/402, 38% in women vs. 110/350, 32%, in men). The follow-up question (asking for thoughts about how to deal with the potential risk) was contingent, requiring two prior positive replies, and no difference was observed between female and male adult offspring (51/151, 41% vs. 451/110, 35%) (Table 4).

**Table 4.** The proportions of male and female adult offspring with heredity for AAA reporting knowledge of relative's disease, awareness of the hereditary risk, and anxiety and depression symptoms via the HADS and EQ-5D instruments. A higher HADS score (≥ 8) is considered as indication of anxiety (A) or depression (D). A higher score on the EQ-5D scale indicates better well-being.

Question 2	Yes	No	Don’t know	Yes	No	Don’t know	*p*-value
Were you aware of the risk to develop AAA as an FDR?	151 (38%)	204 (51%)	44 (11%)	110 (32%	184 (53%)	53 (15%)	0.095
If yes: Have you been experienced anxiety for such a risk?	81 (54%)	64 (42%)	6 (4%)	56 (50%)	54 (49%)	1 (1%)	0.291
If yes: Have you thought about how you could deal with this risk or anxiety?	51 (35%)	78 (53%)	17 (12%)	45 (41%)	58 (53%)	7 (6%)	0.231
HADS and EQ-5D							
HADS total, mean (SD)	6.4 (5.8)			5.4 (5.3)			0.013
HADS-A, mean (SD)	4.2 (3.7)			3.1 (3.3)			<0.001
HADS-D, mean (SD)	2.3 (2.6)			2.3 (2.6)			0.850
HADS-A score ≥ 8	65 (17%)			37 (11%)			0.025
HADS-D score ≥ 8	19 (5%)			15 (4%)			0.861
EQ-5D index score, mean (SD)^a^	6.8 (2.3)			6.4 (2.0)			0.020
EQ-5D VAS 0–100 (SD)	78.9 (17.3)			79.2 (15.0)			0.783

HADS: Hospital Anxiety and Depression Scale; EQ-5D: EuroQol Five Dimension Scale; VAS: visual analogue scale; SD: standard deviation.

^a^ EQ-5D: min 5, max 25.

**Figure 2.**
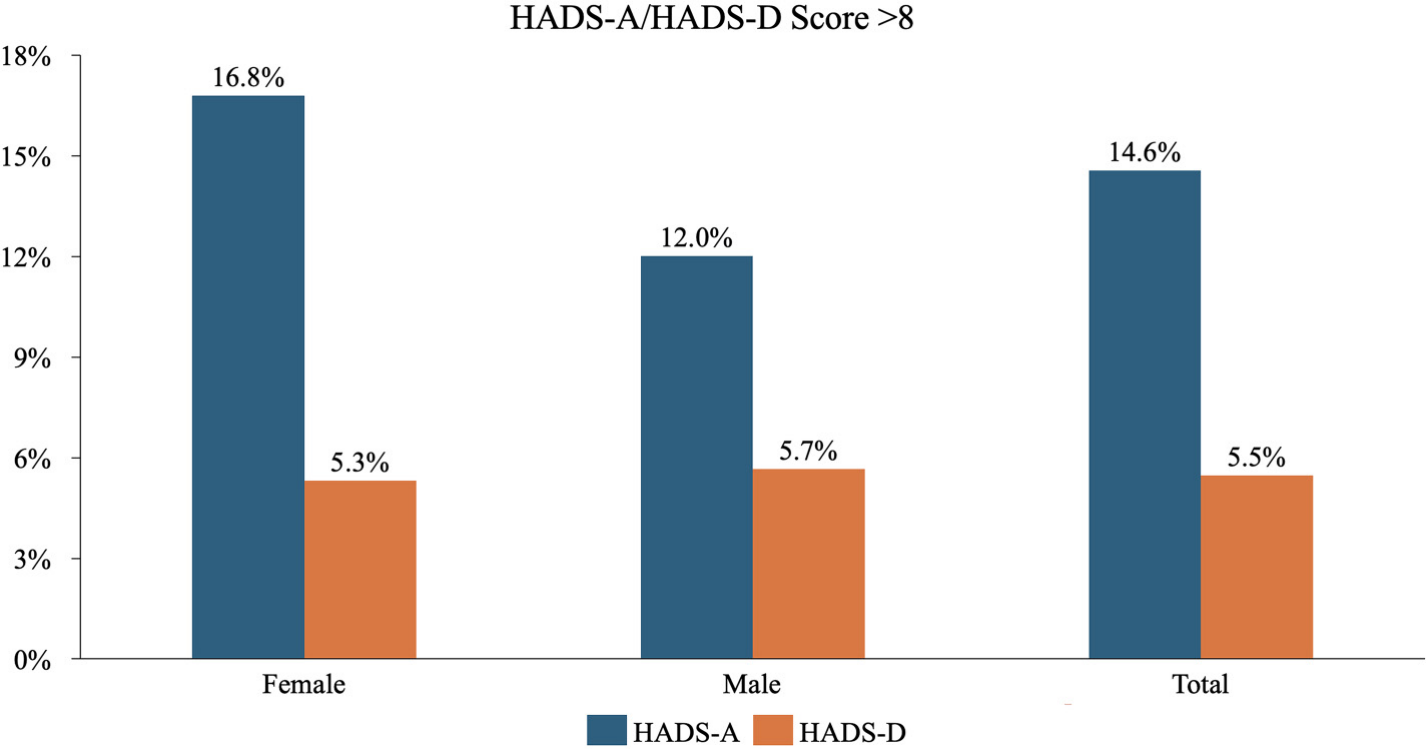
The proportion of individuals with a hospital anxiety and depression scale (HADS-A/D) score (≥ 8): was female and male, and on an aggregated level for all participants.

The female adult offspring had higher HADS and HADS-A mean scores (Table 4). More women reported a summarized HADS-A score over 8 than men (17% vs. 11%, *p* = 0.025) and the EQ-5D index score was higher ((6.8 (2.3) vs. 6.4 (2.0) *p* = 0.020)) for women compared to men (Figure 2) (Table 4). None of the participants reported non-binary gender identity.

### Aortic pathology

In a stratified analysis of participants with AP (*n* = 22) and normal aortic diameters (*n* = 1486), the reported knowledge of an FDR was similar (7/22 vs. 526/1486, respectively) (Supplemental Table S3).

The awareness of the hereditary risk was also similar. The findings via EQ-5D and HADS were similar between the groups, apart from a higher mean of EQ-5D VAS in the AP cohort (71.2 (SD 19.5)) compared to the cohort of normal diameters (79.1 (SD 16)).

## Discussion

The hereditary risk for AAA is well known among vascular surgeons, and the results of this study among examined adult offspring of AAA patients reveal that a large proportion of them are, in fact, aware of their inherent risk of developing the disease. Almost one-fifth also report anxiety associated with this knowledge, although this awareness-related anxiety is not displayed as anxiety symptoms captured by generic instruments. The awareness and anxiety about heritability for AAA among individuals with heredity was, as expected, higher than in the general population. Another perspective is of course that > 60% were not aware of the hereditary risk to develop such a fatal, but treatable, disease. The groups are quite similar to the cohorts from the SCAPIS (Swedish Cardiopulmonary Bioimage Study), a population-based screening of 35,000 persons in Sweden, which provides transferability to the national level and countries with similar demographics.^24^

The high participation rate among those with heredity as compared to controls could primarily be considered as a limitation of the present study. However, the lower participation rate could be considered as a surrogate marker for the awareness and perceived anxiety about a hereditary risk. The difference in participation rate could have introduced a selection bias with differences in characteristics among participants and non-participants.^25^ On the other hand, it is not evident that individuals who are already aware of hereditary risk participate more in screening programs. Experiencing anxiety might also introduce avoidance of screening programs as a strategy. One example has been reported in lung cancer screening, where current smokers are more prone to decline the invitation.^26^ Participation rate could also be influenced by differences in socioeconomic status, which was not evaluated among the participants and non-participants.

The general awareness in the population regarding the hereditary traits and increased risk of developing AAA is hitherto unexplored. In this report, the adult offspring had a higher awareness of heritability, and as expected a higher proportion reported anxiety than among the controls. In a previous study on siblings to AAA patients, 11% reported awareness of heritability, but without comparing controls.^3^ The participants in the sibling study were fewer than in this DAAAD cohort. Knowledge of diseases within families could be higher among children than siblings in general, but the high number of events with emergent aortic diseases reported could of course influence the awareness and anxiety in the adult children.

Notably, our results show that 52% of adult offspring who are aware of the heritability also experience anxiety. In other groups with hereditary traits, such as BRCA carriers, HRQoL has been investigated, and clinically significant anxiety levels were reported 12 months after the confirmation of the BRCA gene.^27^ Consideration of the potential anxiety when communicating with BRCA carriers has been reported to have a positive effect.^28,29^ Hence, this approach might also be applicable when in contact with individuals with a hereditary predisposition for AAA. For adult offspring, the transferability of designated interventions providing psychosocial support should be further explored.^30,31^

The current strategy of relying on patients to inform their families of the screening recommendations for FDRs is clearly insufficient. Furthermore, those who were aware of their risk of developing the disease stated that they had been worried about this risk. It could be interpreted as a failure that more than half of the adult offspring of patients with a potentially fatal and hereditary disease are unaware of the risk of developing a disease which is treatable with elective repair. These results must be reflected upon in adjunct to the recently reported generally lower-than-expected prevalence rate of AAA nationally.^32^ The low prevalence among screened men and in the DAAAD study for women and men in the population with and without heredity, the new European guidelines, and the nationally reported prevalence in the UK: all these reports support that targeted screening is presumably the most cost-effective future national strategy.^16,32–34^ Adult offspring should be informed of their increased risk, but it is important to include balanced information in communication material, such as that risk is also influenced by smoking habits, hyperlipidemia and age of the index person when diagnosed.

The use of questionnaires rather than interviews, in this hitherto unexplored area, was intended to serve as a basis for the design of future investigations into the development of possible communication materials, with qualitative studies exploring the values, knowledge and attitudes towards screening of FDRs.

The dimensionality of the generic HADS instrument is not comparable to the study-specific questions, in that the HADS instrument provides more robust assessments of anxiety and depression symptoms. The HADS also incorporates a time aspect. One recommendation to provide for reliable and valid assessments regarding the impact of AAA disease on HRQoL is to combine generic and disease-specific instruments.^35^ In a general population, approximately 10% score ≥ 8 on HADS^36^ and our results were consistent with this level among the male population. Importantly, the participants are exposed to the questionnaires before the ultrasound examination is performed. Hence, the study design eliminates the possible recall bias that otherwise might influence the results, especially a positive finding of AP.

Women have been scarcely studied with regard to HRQoL in relation to AAA; previous research focused mostly on men participating in population-based screening programs.^11,37^ This study reports contemporary research on HRQoL in women exposed to a targeted screening program. The lower HRQoL levels among the female adult offspring compared to male individuals correspond well with prior reports on sex differences in the population.^38^

It has been reported that men who are diagnosed later with AAA within screening programs rate their general state of health lower than men with normal aortic diameters, even when questionnaires are completed prior to AAA diagnosis.^14,39^ The lower general health has been reported to depend on the commonly detected higher cardiovascular disease burden in patients with AP than in controls.^40^ In this study, the similar cardiovascular risk profiles between the groups, the relatively low mean age, as well as the limited number of persons with AP, could contribute to the lack of disparities in HRQoL. The moderately increased higher self-rated state of health in the participants with AP compared to persons with a normal aortic diameter (measured by EQ-5D VAS) should be interpreted with caution due to the lack of other detected differences in the HRQoL tools.

## Conclusion

This is to date the largest study to explore awareness and anxiety in individuals with a hereditary risk for a potentially lethal disease, AAA. The higher-than-expected proportion of adult offspring with awareness of heritability and anxiety about such risk calls for the development of dedicated strategies to improve communication of AAA risk to patients and their next-of-kin within the healthcare system channels of information.

Communication models must be evaluated in parallel with the actual risk for disease development, preferably in dialogue with patient groups. Our data also generate new basic information on the effect on HRQoL of invitation to selected screening in women and men. The identified anxiety must be further explored using a qualitative study setting in order to understand the full practical implementation of this new knowledge.

## Supplemental Material

sj-docx-1-msc-10.1177_09691413241278224 - Supplemental material for Anxiety and disease awareness in individuals with heredity for abdominal aortic aneurysm Supplemental material, sj-docx-1-msc-10.1177_09691413241278224 for Anxiety and disease awareness in individuals with heredity for abdominal aortic aneurysm by Nina Fattahi, Olga Nilsson, Sverker Svensjö, Joy Roy, Anneli Linné and Rebecka Hultgren in Journal of Medical Screening
