# Supplementary material for: Anxiety and disease awareness in individuals with heredity for abdominal aortic aneurysm
Source: J Med Screen. 2024 Sep 9;32(2):67–75. doi: 10.1177/09691413241278224 (PMC12106935; doi:10.1177/09691413241278224)
Supplement: sj-docx-1-msc-10.1177_09691413241278224 - Supplemental material for Anxiety and disease awareness in individuals with heredity for abdominal aortic aneurysm [file sj-docx-1-msc-10.1177_09691413241278224.docx]

**Supplemental Materials**

**Table S1.** *Two additional study-specific questions were posed (column 1-2). In Table S1 the two specific main awareness questions and following anxiety sub-questions are presented. Questions posed and secondly a “think aloud” summary on the two questions.*

| Questions |  |  |  | Face validity | Content validity |
| --- | --- | --- | --- | --- | --- |
| 1.a | Do you have a close relative with AAA? | | | 7/8 | 8/8 |
| 1b. | **If yes:** Did this person have an acute aneurysm incident (rupture) caused by this diagnosis? | | | 7/8 | 8/8 |
| 2.a | Were you aware of the risk of developing AAA as an FDR? | | | 7/8 | 8/8 |
| 2.b | **If yes**: Have you experienced anxiety about such risk? | | | 8/8 | 8/8 |
| 2.c | **If yes**: Have you been wondering about how to handle this risk or anxiety? | | | 8/8 | 7/8 |

**Table S2.** *The proportion of examined adult offspring and matched controls reporting their knowledge of a next-of-kin with aneurysm disease, their awareness of the hereditary risk. Anxiety and depression symptoms were reported by the HADS and EQ-5D-5L instruments.*

| Women and Men | Adult offspring n= 752 | | | Controls n= 756 | | |  |
| --- | --- | --- | --- | --- | --- | --- | --- |
| Question 1 | **Yes** | **No** | **Don’t know** | **Yes** | **No** | **Don’t know** | **P-value** |
| Do you have a close relative with AAA? | 490 (65%) | 97 (13%) | 164 (22%) | 42 (6 %) | 388 (51%) | 324 (43%) | 0.001 |
| If yes: Did this person had an acute aneurysm incident (rupture) caused by this diagnosis? | 236 (48%) | 195 (40%) | 59 (12%) | 26 (61%) | 11 (26%) | 6 (14%) | 0.195 |
| Question 2 | **Yes** | **No** | **Don’t know** | **Yes** | **No** | **Don’t know** | **P-value** |
| Were you aware of the risk to develop AAA as an FDR? | 261 (35%) | 388 (52%) | 97 (13%) | 78 (10%) | 534 (71%) | 139 (19%) | 0.001 |
| If yes: Have you experienced anxiety for such a risk | 137 (52%) | 118 (45%) | 7 (3%) | 13 (17%) | 59 (75%) | 7 (9%) | 0.001 |
| If yes: Have you thought about how you could deal with this risk or anxiety | 96 (38%) | 136 (53%) | 24 (9%) | 18 (23%) | 51 (65%) | 10 (13%) | 0.052 |
| HADS and EQ-5D |  |  |  |  |  |  |  |
| HADS total, mean (SD) | 5.9 (5.6) | | | 6.5 (5.7) | | | 0.039 |
| HADS-A, mean (SD) | 3.7 (3.6) | | | 4.0 (3.4) | | | 0.071 |
| HADS- D, mean (SD) | 2.3 (2.6) | | | 2.6 (2.8) | | | 0.031 |
| HADS A score $\boldsymbol{\geq}$8 | 102 (14%) | | | 114 (15%) | | | 0.419 |
| HADS D score $\boldsymbol{\geq}$8 | 34 (4.6%) | | | 47 (6.4%) | | | 0.139 |
| EQ 5D index score, mean (SD)* | 6.6 (2.2) | | | 6.8 (2.3) | | | 0.113 |
| EQ 5D VAS scale 0-100 | 79 (16.3) | | | 79 (16.0) | | | 0.947 |

**Table S3.** *Analysis of the study population stratified for participants with aortic pathology (AP) and participants with normal aortic diameter. Awareness of the hereditary risk, anxiety, and depression symptoms are reported by the HADS and EQ-5D-5L instruments. A higher HADS-score (≥8) is considered as indication of Anxiety or Depression. A higher score on the EQ-5D scale indicates better well-being.*

| Women and Men | Aortic Pathology n=22 | | | Normal aortic diameter n= 1486 | | |  |
| --- | --- | --- | --- | --- | --- | --- | --- |
| Question 1 | **Yes** | **No** | **Don’t know** | **Yes** | **No** | **Don’t know** | **P-value** |
| Do you have a close relative with AAA? | 7 (32%) | 11 (50%) | 4 (18%) | 526 (36%) | 474 (32%) | 483 (33%) | 0.172 |
| Question 2 | **Yes** | **No** | **Don’t know** | **Yes** | **No** | **Don’t know** | **P-value** |
| Were you aware of the risk to develop AAA as an FDR? | 3 (14%) | 13 (59%) | 6 (27%) | 336 (23%) | 909 (61%) | 230 (16%) | 0.238 |
| HADS and EQ-5D |  |  |  |  |  |  |  |
| HADS total, median (IQR) | 4.0 (5.0) | | | 5.0 (7.0) | | | 0.417 |
| HADS-A, median (IQR) | 2.0 (3.0) | | | 3.0 (5.0) | | | 0.191 |
| HADS- D, median (IQR) | 1.5 (2.0) | | | 1.0 (2.0) | | | 0.908 |
| HADS A score $\boldsymbol{\geq}$8 | 2 (9 %) | | | 214 (15%) | | | 0.759 |
| HADS D score $\boldsymbol{\geq}$8 | 1 (5 %) | | | 80 (6%) | | | 1.000 |
| EQ 5D index score, median (IQR)* | 6.0 (2.0) | | | 6.0 (2.0) | | | 0.926 |
| EQ 5D VAS scale 0-100, median (IQR) | (79.1 (16.0)) 80.0 (25.0) | | | 71.2 (19.5) 80.0 (18.0) | | | 0.042 |
